# Efficacy of *Limosilactobacillus fermentum* in the management of vulvovaginal candidiasis: comparative analysis with topical miconazole in a single-blind randomized clinical trial

**DOI:** 10.3389/fmicb.2024.1428590

**Published:** 2024-08-01

**Authors:** Marco Pane, Emanuele Chisari

**Affiliations:** Probiotical Research Srl, Novara, Italy

**Keywords:** vulvovaginal candidiasis, probiotics, *Limosilactobacillus fermentum*, miconazole, clinical trial, treatment efficacy, recurrence rate

## Abstract

**Introduction:**

Vulvovaginal candidiasis (VVC) significantly impacts women’s quality of life and often shows a high recurrence rate despite conventional antifungal therapies. This study evaluates the efficacy of *Limosilactobacillus fermentum* (LF5), a probiotic, as an alternative treatment option to conventional miconazole therapy in managing VVC.

**Methods:**

The randomized, single-blind clinical trial involved 100 premenopausal women diagnosed with VVC. Participants were assigned to either a vaginal capsule containing LF5 probiotic strain or miconazole. Treatments were administered once daily for three consecutive days. Microbiological eradication of *Candida* spp. and recurrence rates were assessed at 30 days post-treatment. The trial was registered with the Italian Ministry of Health.

**Results:**

Both treatments achieved a high rate of microbiological eradication of Candida spp. within the three-day treatment period (96% for LF5 and 94% for miconazole). Recurrence rates within 2 weeks post-treatment were low and similar between the groups (10% for LF5 and 17% for miconazole). LF5 was found to have a significantly lower incidence of local adverse reactions compared to miconazole (4 vs. 12%).

**Discussion:**

LF5 presents a viable alternative to miconazole for the treatment of VVC, offering comparable efficacy with fewer side effects. The results suggest that probiotic treatments can potentially enhance patient compliance and quality of life by reducing adverse reactions and recurrence rates. Further research is needed to confirm these findings in larger and more diverse populations.

## 1 Introduction

Vulvovaginal candidiasis (VVC) is one of the most common vaginal conditions affecting women worldwide and the second most common cause of vaginal infection in the United States (Sobel, 2007; Nyirjesy et al., 2022). It is characterized by a disruption in the vaginal microbiota, shifting from a lactobacilli-dominated environment to a more diverse microbial ecosystem (Sun et al., 2023), which becomes permissive to fungi part of the *Candida* spp. such as *C. albicans* or *C. glabrata* (Sobel, 2007; Nyirjesy et al., 2022) This condition is clinically significant not only because it affects a substantial portion of the female population worldwide, but also due to its severe influence on the general wellbeing and quality of life of the affected women and its high recurrence rate (Fukazawa et al., 2019; Lietz et al., 2023).

A limited selection of antifungal treatments is available for this condition, mainly based on the azoles family and nystatin for both topical and/or systemic therapy (Cochrane STI Group et al., 2022). Standard-of-care treatment is able to successfully treat VVC acutely (Cochrane STI Group et al., 2022; Nyirjesy et al., 2022) and in chronic cases (Lietz et al., 2023). However, traditional antifungal treatments face growing challenges such as drug resistance (Fisher et al., 2022; Vitiello et al., 2023) and toxicity (Sobel, 2007; Nyirjesy et al., 2022) because fungal cells share many structural similarities with human cells, which has made selective targeting difficult without harming the host (Ostrosky-Zeichner et al., 2010). On the contrary, probiotics offer a less invasive option that can reduce the incidence of side effects (Sanders et al., 2010) and the emergence of drug-resistant fungal strains, aligning with the holistic One Health approach (A One Health Approach to Combating Fungal Disease: Forward-Reaching Recommendations for Raising Awareness, 2019) that emphasizes integrated health strategies across humans, animals, and the environment. Side effects and drug resistance emphasize the need for careful treatment of VVC and consideration of alternative treatment options in affected individuals.

Probiotics have emerged as a promising adjunct or alternative therapy to antimycotics for the treatment of VVC (Falagas et al., 2006; Xie et al., 2017; López-Moreno and Aguilera, 2021). In particular, there is significant interest behind the capacity of probiotics to not only to acutely manage the disease but favor a vaginal environment that is hostile to colonization by *Candida* spp., thus potentially reducing the recurrence rate (Nyirjesy et al., 2022). Recurrence rates following antimycotic treatment are high, with more than 25% of women experiencing a recurrence within 1 month after testing negative for *Candida* spp. and more to recur in subsequent months (Sobel, 2007). This high recurrence rate suggests an underlying limitation in the antimycotic treatment’s ability to restore the beneficial lactobacilli-dominated vaginal flora (Sun et al., 2023) or disrupt the vaginal and extra-vaginal reservoir of infection (e.g., intestinal presence of *Candida* spp.) (Nystatin Multicenter Study Group, 1986; Novikova et al., 2002; Reed et al., 2003; Jensen et al., 2024). Still, great heterogeneity in evidence quality and strain characteristics limit the adoption of probiotics as an alternative to current treatment options, which warrants further research in this space.

Large-scale randomized controlled trials are necessary to confirm the benefits observed in smaller preliminary studies and to establish guidelines for the use of probiotics in the treatment of VVC, particularly in diverse populations. This holistic approach to VVC could potentially lead to significant improvements in women’s reproductive health globally.

## 2 Materials and methods

### 2.1 Study design

This study employed a randomized, single-blind, controlled design to evaluate the efficacy of a vaginal probiotic treatment compared to standard-of-care miconazole therapy in premenopausal women diagnosed with VVC. The treatment regimen consisted of a once a day probiotic vaginal capsule taken for 3 consecutive days. Each capsule contained 1 × 10^^9^ CFU of *Limosilactobacillus fermentum* LF5 (I-789). The primary objective was to assess the impact of probiotic supplementation on VVC cure rates, assessed through microbiological assessment at 3 days after treatment. The study was approved by the Italian Ministry of Health on 8 March 1988 (Supplementary material 1) and it was concluded in June 1992.

### 2.2 Probiotic strain identification

*Limosilactibacillus fermentum* LF5 (I-789), isolated from the vaginal habitat, was identified using genetic assays to determine the guanine-cytosine content of its genomic DNA and through hybridization assays with the DNA of the reference strain *Limosilactibacillus fermentum* ATCC 14932. The isolation of the *Limosilactibacillus fermentum* LF5 strain from the vaginal microflora of a healthy subject was carried out by Tosi Farmaceutici Srl. (Novara, Italy). The strain has been deposited at the International Bacterial Collection of the Pasteur Institute in Paris under accession number I-789.

### 2.3 Participants

Inclusion criteria included microbiological diagnosis of VVC based on traditional culture exam, and ability to provide informed consent. Exclusion criteria included hypersensitivity to any of the ingredient used in the two formulations, unable to provide consent or already involved in other clinical studies within the prior 30 days. The study included 100 women aged 19–61 years, all diagnosed VVC. They were identified based on clinical symptoms, specifically vaginal irritation and discharge. The diagnosis was confirmed by microbiological culture for *Candida* spp.

### 2.4 Intervention

Participants were randomized into two groups. The treatment group received either a vaginal capsule containing LF5 probiotic strain (109 CFUs/dose) or miconazole (400 mg/dose) once a day before sleep (Supplementary material 2 for the full pharmaceutical composition). Both the probiotic and miconazole treatments were administered daily for a duration of 3 days. All participants were assessed for 2 weeks after the end of the treatment period.

### 2.5 Randomization and blinding

The randomization was generated on computer and then provided as a table to the experimental team. To maintain the integrity of the study, participants were blinded to group assignments. The probiotics and placebo treatments were identically packaged to ensure that blinding was effective.

### 2.6 Outcome measures

The primary outcome measure was VVC resolution, defined by microbiological eradication of *Candida* spp. at 3 days. Secondary outcomes included persistent cure rate 2 weeks after treatment, self-reported symptomatology and physician-assessed presence of epithelization, erythema, and purulent discharge, all tested at baseline, 1, 2, 3 days, and at the end of the study (2 weeks). The self-reported and physician-assessed scoring was based on a Linkert-like scale where 0 means absent and 3 meant severe.

### 2.7 Culture methodology

Following sample collection, the specimens were delivered to the hospital laboratory within a 2-h window. Upon appropriate inspection and processing, the samples were inoculated onto Sabouraud agar supplemented with chloramphenicol and subsequently incubated at 37°C for 72 h. Identification of colonies belonging to the genus *Candida* was achieved through both macroscopic and microscopic examination. Species-level identification was not performed.

### 2.8 Statistical methods

Data were analyzed with intent to treat. The difference in cure rates between the probiotic and placebo groups was evaluated using the Chi-square test for independence. A *p* < 0.05 was considered statistically significant.

## 3 Results

A total of 100 patients diagnosed with VVC were included in this study. No significant differences were found between the interventional and control treated cohorts for age (38.6 ± 1.85 vs. 37.3 ± 1.77, *p* = 0.49) at the time of enrollment.

**Figure 1:**
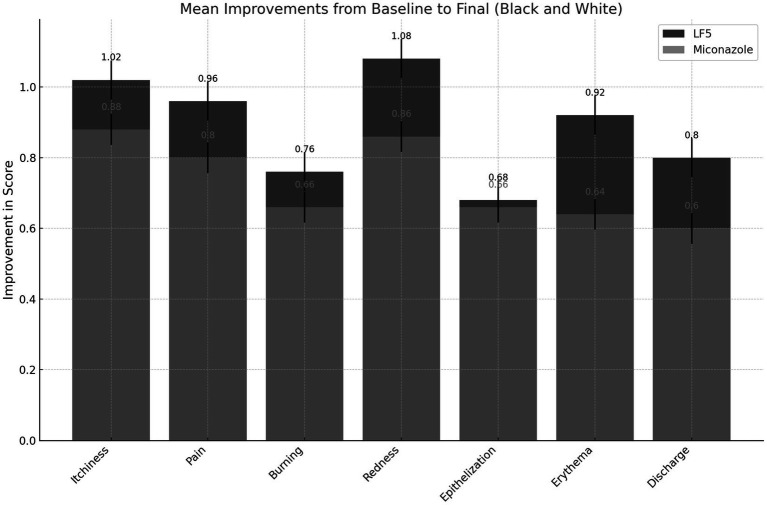
Mean improvements in self-reported patient outcomes. Data collection: scores for each parameter were recorded at various time points (Baseline, Day 1, Day 2, Day 3, and Final) for both LF5 and Miconazole treatments. Improvement calculation: the mean improvement for each parameter was calculated as the difference between the baseline and final scores. The standard error of the mean (SEM) was computed to provide error bars representing the variability of the data.

Both treatments achieved microbiological eradication of *Candida* in almost all patients at the end of the 3-day treatment period (96 vs. 94%). Furthermore, the risk of recurrence within the 2 weeks after treatment was very low for both treatments. Among the patients who achieved microbiological cure, the risk of recurrence was similar with miconazole (17%, 8/47 patients) compared to LF5 (10%, 5/48 patients) (*p* = 0.372). Symptomatic remission was also very favorable with both treatments (Figure 1).

Thus, in general, LF5 is confirmed to be at least as clinically potent as the reference drug, capable of producing appreciable, and statistically significant results that are globally beneficial for patients (Table 1). Medical evaluation at the end of the observation period regarding clinical efficacy is consistent with the observed trends in both self-reported symptomatology (Table 1) and microbiology, which is notably is the most discriminating element in this study.

**Table 1:** Summary of the patient-self-reported and physician-assessment outcome throughout the study.

Parameter	Timepoint	LF5	Miconazole	Mann–Whitney *p*-value
Ichness	Baseline	1.18 ± 0.12	1,100 ± 0.12	1.186
	Day 1	0.84 ± 0.08	0.78 ± 0.09	0.317
	Day 2	0.78 ± 0.07	0.72 ± 0.08	0.409
	Day 3	0.08 ± 0.05	0.08 ± 0.05	0.000
	Final	0.16 ± 0.07	0.22 ± 0.08	0.693
Pain	Baseline	1.08 ± 0.12	1.02 ± 0.10	0.185
	Day 1	0.74 ± 0.07	0.82 ± 0.07	0.124
	Day2	0.72 ± 0.07	0.80 ± 0.07	0.617
	Day3	0.04 ± 0.04	0.08 ± 0.05	0.990
	Final	0.12 ± 0.05	0.22 ± 0.08	0.859
Burning	Baseline	0.90 ± 0.12	0.88 ± 0.12	0.008
	Day 1	0.62 ± 0.08	0.66 ± 0.08	0.091
	Day 2	0.60 ± 0.08	0.62 ± 0.07	0.038
	Day 3	0.06 ± 0.04	0.08 ± 0.05	0.200
	Final	0.14 ± 0.06	0.22 ± 0.08	0.774
Redness	Baseline	1.20 ± 0.12	1.12 ± 0.11	0.297
	Day 1	0.60 ± 0.09	0.58 ± 0.10	0.237
	Day2	0.56 ± 0.08	0.54 ± 0.10	0.283
	Day 3	0.04 ± 0.04	0.14 ± 0.06	2.722
	Final	0.12 ± 0.05	0.26 ± 0.09	0.972
Epithelization	Baseline	0.84 ± 0.12	0.86 ± 0.12	0.011
	Day 1	0.68 ± 0.09	0.64 ± 0.08	0.050
	Day 2	0.64 ± 0.08	0.62 ± 0.08	0.013
	Day 3	0.10 ± 0.06	0.08 ± 0.06	0.192
	Final	0.16 ± 0.07	0.20 ± 0. 07	0.1645
Erythema	Baseline	1.10 ± 0.11	0.92 ± 0.12	1.228
	Day 1	0.88 ± 0.09	0.72 ± 0.09	1.658
	Day 2	0.82 ± 0.08	0.70 ± 0.09	1.205
	Day 3	0.10 ± 0.05	0.12 ± 0.05	0.388
	Final	0.18 ± 0.08	0.28 ± 0.09	0.764
Discharge	Baseline	0.96 ± 0.11	0.84 ± 0.12	0.594
	Day 1	0.74 ± 0.08	0.62 ± 0.09	1.139
	Day 2	0.70 ± 0.08	0.60 ± 0.09	1.304
	Day 3	0.08 ± 0.05	0.10 ± 0.05	0.146
	Final	0.1610107	0.24 ± 0.08	0.743
Total of symptoms and signs	Baseline	7.26 ± 0.38	6.64 ± 0.28	0.619
	Day 1	5.10 ± 0.33	4.82 ± 0.29	0.229
	Day 2	4.82 ± 0.26	4.60 ± 0.24	0.269
	Day 3	0.50 ± 0.28	0.68 ± 0.27	0.773
	Final	1 0.04 ± 045	1 0.64 ± 0.54	0.783

All patients completed the treatment cycle as expected. The clinical tolerability was good for both preparations. However, the frequency of local adverse experiences was three times higher with miconazole (12%, six cases) compared to LF5 (4%, two cases). All adverse events were mild to moderate in severity (see Supplementary material 3).

The risk of intolerance, while of minimal clinical significance in terms of nature and intensity, was consistent with the known literature (Sobel, 2007; Cochrane STI Group et al., 2022) for miconazole at 12% (95% CI, 3–21). The incidence of adverse experiences with LF5 was approximately one third that observed with miconazole, at 4% (95% CI, 0–9).

The symptoms and signs reported as adverse reactions can be interpreted in both cases as indicators of mild local intolerance due to the expected pharmacodynamic action: in the case of LF5, this is probably due to local acidification following the *in situ* release of the live cultures and their metabolic activity. They can also be viewed as a modest indicator of intolerance to the application of the vaginal capsule itself even though the evidence gathered does not support inflammatory or immune reaction to any of the components. However, the minor nature and the rapid spontaneous reversibility of the reported reactions guarantee good local tolerability of both formulations examined, with LF5 offering a noticeable advantage in terms of risk. No signs of systemic adverse reactions or potential secondary pharmacodynamic actions were observed.

Similarly, the trend in the hematological and biochemical safety tests indicates the absence of negative effects of the treatments on the parameters considered. In general, there are no signs of potential interference from treatments on the evolution of hematological and biochemical measures.

## 4 Discussion

This study highlights the potential of LF5 probiotic treatment as an effective alternative to standard miconazole therapy for the treatment of vulvovaginal candidiasis (VVC), with the dual benefits of achieving comparable clinical efficacy and reducing adverse reactions. As vulvovaginal candidiasis remains a major challenge in women’s health, marked by high rates of recurrence and significant discomfort, our findings provide valuable insights into possible improvements in therapeutic strategies.

The results of this study are significant, showing that the use of the LF5 strain not only matched the antimycotic effects of miconazole, but also exhibited a lower incidence of local adverse reactions. Specifically, LF5 demonstrated a three-fold reduction in adverse experiences compared to miconazole, with only 4% of participants experiencing mild to moderate symptoms. This favorable safety profile underscores the importance of considering patient tolerance and side effects in VVC treatment, a perspective supported by similar findings in previous research where probiotic use was associated with minimal side effects. Our findings are further supported by the preexisting preclinical and clinical evidence available for *L. fermentum*, which shows activity against *Candida* spp. previously published by our group (Vicariotto et al., 2012; Murina et al., 2014; Deidda et al., 2016a,b).

Compared to the existing literature, the existing literature provides a strong foundation for our study (Vicariotto et al., 2012; Murina et al., 2014; Deidda et al., 2016a,b). Traditional VVC management often relies on antimycotics such as miconazole, but the recurrence rate remains high, pointing to the limitations of these treatments in restoring the normal vaginal microbiota (Cochrane STI Group et al., 2022; Nyirjesy et al., 2022). Our study supports the hypothesis that probiotics can be a crucial adjunct, not just in managing symptoms but in potentially altering the course of the disease by stabilizing the vaginal flora (Falagas et al., 2006; Xie et al., 2017; López-Moreno and Aguilera, 2021). This aligns with the work by Sun et al. (2023) who noted that a Lactobacillus-dominated microbiota could prevent the overgrowth of pathogenic fungi.

The practical implications of our research are profound. By reducing the risk of recurrence and minimizing side effects, probiotic-based treatment could improve patient compliance and quality of life. This approach could also reduce the need for repeated antimycotic treatments, which often lead to resistance and additional complications. Theoretically, the establishment of a robust vaginal microbiota could also offer long-term protective effects against other vaginal infections, although this remains to be explored in future studies.

However, despite these promising results, our study is not without limitations. The sample size, while adequate for initial findings, is relatively small for making generalized conclusions. Additionally, the variability in probiotic strains and treatment regimens in different studies makes it difficult to recommend a standardized protocol. The heterogeneity in evidence quality noted in our results suggests that larger, more comprehensive trials are necessary to validate these findings in diverse populations. Last but not least, our study was limited to the 2-week time point which may have limited our capacity to identify VVC cases happening at a later date resulting in false negatives. However, for our study design, we considered this observation period acceptable.

In the future, it is crucial to explore the long-term effects of probiotic treatment on the vaginal microbiota and its impact on recurrence rates. Further research should also investigate the optimal types and doses of probiotics and whether different strains might offer better results. Establishing clear guidelines for the use of probiotics in VVC treatment will be essential, especially considering the varying susceptibilities of different demographic groups.

## 5 Conclusion

This study illustrates that probiotic treatment, particularly using the LF5 strain, is a viable alternative to traditional antimycotic therapy for VVC, offering comparable efficacy with fewer side effects. The journey of understanding and treating VVC has been substantially advanced by integrating probiotics into therapeutic regimens. This approach not only addresses the immediate effects of infection, but also contributes to the broader goal of enhancing women’s reproductive health globally. The promising results of this study set the stage for a change in the management of vulvovaginal candidiasis, focusing on the role of microbiota stability in the achievement of long-term health outcomes.

## Data availability statement

The raw data supporting the conclusions of this article will be made available by the authors, without undue reservation.

## Ethics statement

The studies involving humans were approved by the clinical study was conducted by Dr. Antonio Iannino, Ente Ospedaliero Di Civitanova (Italy, Marche) under local Ethical Committee. The product was registered as a drug on the Italian market by Tosi Farmaceutici (Italy, Novara) with the name LAB/A (A.I.C. N. 028974018) and approved for commercialization the 6th of July 1998 by the Italian Health Authority (NCR n. 297). The studies were conducted in accordance with the local legislation and institutional requirements. The participants provided their written informed consent to participate in this study.

## Author contributions

MP: Writing – review & editing, Writing – original draft, Visualization, Supervision, Methodology, Investigation, Formal analysis, Data curation. EC: Writing – review & editing, Writing – original draft, Supervision, Methodology, Investigation, Formal analysis, Data curation.
