# Supplementary material for: Efficacy of Limosilactobacillus fermentum in the management of vulvovaginal candidiasis: comparative analysis with topical miconazole in a single-blind randomized clinical trial
Source: Front Microbiol. 2024 Aug 1;15:1428590. doi: 10.3389/fmicb.2024.1428590 (PMC11324542; doi:10.3389/fmicb.2024.1428590)
Supplement: Supplementary file 1 [file Data_Sheet_1.pdf]

The Italian text extracted from the image translates to English as follows:

Date 8th of March 1988

Ministry of Health

Direction for general pharmaceutical service

Addressed to Tosi Farmaceutici Srl, Corso della Vittoria 12, Novara

Subject: Pharmaceutical product TF 88.1

Composition: Vaginal capsul NLT (no less than) $1x10^6$ cells for vaginal use of LF5 (L. fermentum).

"We communicate that, having examined the application indicated in the subject, according to circular no. 35 of April 9, 1975, this Ministry considers that the above-mentioned product must be considered not of new establishment, and therefore not subject to the checks prescribed by art. 1, paragraph two, letter i) of the law of August 7, 1973, no. 519, as products with similar composition, quantity, method of administration, and dosage have already been recognized not of new institution in Italy with similar composition qualitatively and quantitatively..

It is communicated, however, that with letter n 800. MON.AG.695/945 as of June 30, 1986, addressed to Farmindustria, instructions have been issued regarding the implementation of pharmaceutical products, noting the provisions provided by Decrees DD.MM 20.3.1980 and of January 27, 1984, to products under clinical development.

In offering the aforementioned provisions, we require your company to communicate, under its responsibility, any adverse effects that occur during the use of the indicated pharmaceutical product."

The general director

Signed

MODULARIO SANITÀ 5

MOD. 7 - U G.

Roma, 8.3.1988

Ministero della Sanità
Direzione Generale Servizio
Farmaceutico
N. 800 ~~MM~~/C.35.757/3082
Risposta al Foglio del
N.

Alla Ditta
Tosi Farmaceutici S.r.l.
Corso della Vittoria, 12/B
Novara

OGGETTO: Prodotto farmaceutico TF 88.1

capsule vaginali N.L.T. 1.10⁶ cellu

Composizione flaconi e flaconcini di lavanda pronta uso vaginale N.L.T. 1.10⁶ cellule di LFS (fermenti

Si comunica che, esaminata la domanda indicata in oggetto ai sensi della circ.n. 35 del 9 aprile 1975, questo Ministero ritiene che il sopraindicato prodotto deve essere considerato non di nuova istituzione e, pertanto, non soggetto agli accertamenti prescritti dall'art. 1- comma secondo- lettera l) della legge 7 agosto 1973, N° 519, in quanto già sono stati riconosciuti non di nuova istituzione in Italia prodotti con analoga composizione quali -quantitativa, via di somministrazione e dosaggio.

Si comunica, tuttavia, che con lettera n. 800.MON.AG.695/945 del 30.6.1986, indirizzata alla Farmindustria, sono state impartite disposizioni finalizzate ad un sempre più efficace controllo della sicurezza di impiego dei prodotti farmaceutici e, in particolare, sono stati estesi gli adempimenti previsti dai DD.MM. 20.3.1980 e 28.7.1984 ai prodotti in corso di sperimentazione clinica.

Nel confermare le suddette disposizioni e nel richiamare la scrupolosa osservanza delle stesse, si invita codesta Ditta a comunicare, sotto la sua responsabilità, ogni effetto indesiderato che si verifichi nel corso dell'eventuale sperimentazione del prodotto farmaceutico indicato in oggetto.

IL DIRETTORE GENERALE
