# Supplementary material for: Efficacy of Limosilactobacillus fermentum in the management of vulvovaginal candidiasis: comparative analysis with topical miconazole in a single-blind randomized clinical trial
Source: Front Microbiol. 2024 Aug 1;15:1428590. doi: 10.3389/fmicb.2024.1428590 (PMC11324542; doi:10.3389/fmicb.2024.1428590)
Supplement: Supplementary file 2 [file Data_Sheet_2.pdf]

Composition of LF5 - vaginal capsules

- Each vaginal capsule contains:
- Active principle:
    - Lactobacillus Fermentum(LF5) I-789 freeze-dried
- Excipients:
    - Medium chain triglycerides
    - Silica(Aerosil 300)
    - Composition of the casing
    - Gelatin F.U.
    - Glycerol F.U.
    - Dimethylposiloxane 1000
    - Titanium Dioxide E 171
