# Supplementary material for: Efficacy of Limosilactobacillus fermentum in the management of vulvovaginal candidiasis: comparative analysis with topical miconazole in a single-blind randomized clinical trial
Source: Front Microbiol. 2024 Aug 1;15:1428590. doi: 10.3389/fmicb.2024.1428590 (PMC11324542; doi:10.3389/fmicb.2024.1428590)
Supplement: Supplementary file 3 [file Data_Sheet_3.pdf]

Table 1. Adverse event description

ID No.	Event description
**Treatment Group: LF5**	
69	Moderate burning sensation for approx. 30 minutes after application of the first two capsules, it disappeared spontaneously
82	minimal local irritation reaction upon application of the first capsule, duration approx. 2 h, disappeared spontaneously
**Treatment Group: Miconazole**	
7	slight burning sensation upon introduction of 118 capsules, duration approx. 15 min disappeared spontaneously
32	moderate sense of irritation upon application of the capsules with redness lasting approximately 2 hours, spontaneously reversible
58	moderate burning for approx. 1.5 hours and slight redness until the morning after the application of the capsules, spontaneously reversible, probably related to the treatment.
71	slight sense of vaginal heaviness upon application of the capsules lasting approx. 30 min, disappeared spontaneously.
91	moderate sense of heaviness and slight burning for approx. 30 minutes after the application of the chasubles, they disappeared spontaneously
98	slight sense of irritation and vaginal heaviness upon application from the first capsule, lasting approx. 1 h, disappeared spontaneously, possibly related to treatment.

Table 2. Safety parameters to assess tolerability of the interventions

Parameter	Timepoint	LF5	Miconazole	t—test p-value
Erythrocytes (10E+12/L)	Baseline	4.15±0.02	4.16±0.02	0.394
	Final	4.14±0.03	4.17±0.03	0.705
Hemoglobin (g/dl)	Baseline	12.95±0.10	12.75±0.10	1.528
	Final	12.87±0.12	12.80±0.14	0.435
Leukocytes (10E+9/L)	Baseline	8.22±0.10	8.14±0.07	0.664
	Final	8.16±0.13	8.08±0.09	0.549
AST (U/L)	Baseline	14.76±0.85	13.68±0.76	0.948
	Final	14.44±0.85	13.76±0.78	0.588
ALT (IJ/L)	Baseline	17.62±0.74	17.66±0.84	0.036
	Final	17.52±0.81	17.82±0.90	0.249
Bilirubin (mg/dl)	Baseline	0.74±0.02	0.72±0.02	0.560
	Final	0.74±0.02	0.72±0.02	0.626
Nitrogen levels (mg/dl)	Baseline	37.60±1 .oo	35.78±0.88	1.365

	Final	37.30±1.07	36.14±0.93	0.818
Glycemia (mg/dl)	Baseline	100.12±1.40	102.66±1.23	1.365
	Final	99.42±1.68	103.30±1.37	1.786
Creatinine (mg/dl)	Baseline	0.73±0.01	0.72±0.01	0.750
	Final	0.73±0.01	0.72±0.01	1.197
Alkaline Phosphatase (U/L)	Baseline	96.76±1.39	94.94±1.32	0.949
	Final	95.26±1.57	94.46±1.32	0.390
